# Elucidating simulated equivalence responding through dynamic visualization of structural connectivity and relational density

**DOI:** 10.3389/frai.2025.1618678

**Published:** 2025-08-05

**Authors:** James O'Sullivan, Freddy Jackson Brown, Oliver Ray

**Affiliations:** ^1^Department of Computer Science, University of Bristol, Bristol, United Kingdom; ^2^Centre for Research in Intellectual and Developmental Disabilities, University of Warwick, Coventry, United Kingdom

**Keywords:** Stimulus Equivalence, computational modeling, explainable AI, reinforcement learning, relational density theory, relational frame theory

## Abstract

This article presents Affinity, a visual analytics tool that enhances the simulation of the emergence of derived relations between stimuli in humans. Built on the foundations of a reinforcement learning model called Enhanced Equivalence Projective Simulation, Affinity provides both real-time visualizations of the agent's relational memory and enables the simulation of Relational Density Theory, a novel approach to understanding relational responding through the modeling of higher-order properties of density, volume, and mass. We demonstrate these features in a simulation of a recent study into the quantification of relational volume. We also use this as an opportunity to examine the effect of the underlying model's consolidation mechanism, Network Enhancement, on the agent's relational network. Our results highlight Affinity's innovation as an explainable modeling interface for relational formation and a testbed for new experiments. We discuss the limitations of Affinity in its current state, underline future work on the software and computational modeling of Stimulus Equivalence and locate this contribution in the broader scope of integrations of Contextual Behavioral Science and Artificial Intelligence.

## 1 Introduction

Affinity is a novel tool for exploring the formation of trained and derived relations of stimuli in experimental simulations. By extending a pre-existing computational model called Enhanced Equivalence Projective Simulation (EEPS) (Mofrad et al., 2021), Affinity provides real-time visualizations of an agent's relational development, which, in EEPS, constitutes a network of observations and actions obtained from the environment, in the form of dynamic graph networks and heatmaps that capture the intricacies of relations between stimuli. Additionally, Affinity breaks ground by incorporating Relational Density Theory (RDT) into its analytics. RDT is a novel proposal that conceptualizes networks of related stimuli as having volume, density, and mass (Belisle and Dixon, 2020b). Therefore, this innovative approach allows the software to model the agent's mental state conceptually as a physical system, using various factors of the agent's memory as potential measures of density and volume. Affinity differentiates itself from EEPS with these features, generating unique insights into RDT and bridging a gap between computational simulations in Contextual Behavioral Science and Explainable and Understandable AI.

Described as one of the most studied phenomena in behavioral science, Stimulus Equivalence (SE) describes how conditional discriminations can emerge naturally without reinforcement so long as prior conditional discriminations have been established (Sidman, 1971; Sidman and Tailby, 1982; Green and Saunders, 1998). These prior conditional discriminations are learned in a unidirectional fashion, with reflexivity (A = A) first, then symmetry (if A = B, then B = A) and finally transitivity (if A = B and B = C, then C = A), with the formation of these three discriminations necessary for an individual to perform equivalence responding (Sidman and Tailby, 1982). While SE provided a robust initial framework for exploring language generativity, it is limited to equivalence relations and primarily a descriptive framework of the phenomena that did not explain how individuals acquired these discrimination skills. In later years, Steven C. Hayes and colleagues developed Relational Frame Theory (RFT), proposing humans learn a generalized ability to relate stimuli in flexible, context-dependent ways to form networks of meaning beyond simple equivalence and direct learning histories (Hayes et al., 2001). RFT describes how humans learn to respond in generalized ways to the relationships between stimuli in increasingly arbitrary ways (i.e., the relations between the stimuli are based on socially agreed conventions rather than any physical characteristics of the stimuli themselves).

The experimental precision and scope of SE and RFT in studying language and cognition provide a robust and flexible basis for Artificial Intelligence (AI) researchers aiming to model language and cognitive development (Tovar et al., 2023). The simulation of these theories serves a dual purpose. For behavioral scientists, each model is a pathway for exploring abstract and methodological questions on the theory. For AI researchers, simulating SE presents an opportunity for developing a clearer perspective on how AI models can exhibit understandable, human-like abilities in symbolic learning and perspective-taking (Johansson and Lofthouse, 2023).

Mofrad et al. (2020, 2021) have developed two models of SE in this area called Equivalence Projective Simulation (EPS) and Enhanced Equivalence Projective Simulation (EEPS). These simulacra stand out from other feed-forward and neural network models in the field (Lew et al., 2008; Lew and Zanutto, 2011; Tovar and Chávez, 2012; Ninness et al., 2018) thanks to their focus on reinforcement learning, which is applied through a novel framework called Projective Simulation (PS) (Briegel and De las Cuevas, 2012).

In PS, an agent possesses a memory composed of a directed network of nodes, called clips, that are generated and reconfigured throughout the agent's lifespan:


(1) $$\begin{array}{l} c\equiv \left(c_{1},c_{2},...,c_{n}\right)\in C \end{array}$$

A clip can be created following the perception of some input within a portion of the agent's environment, called the percept space. These inputs or percepts can have several characteristics, such as size, speed, or color, and the percept and its characteristics are recorded within the agent's clip space as a “remembered” version of it, defined as *s*_*i*_:


(2) $$\begin{array}{l} s\equiv \left(s_{1},s_{2},...,s_{m}\right)\in S_{1}\times ...\times S_{m}\equiv S,s_{i}=1,...,|S_{i}| \end{array}$$

Alongside the percept space, an agent has access to an actuator space containing all possible actions an agent can undertake. As with percepts, the agent can create versions of the actuators in their clip space:


(3) $$\begin{array}{l} a\equiv \left(a_{1},a_{2},...,a_{p}\right)\in A_{1}\times ...\times A_{p}\equiv A,a_{j}=1,...,|A_{j}| \end{array}$$

**Figure 1:**
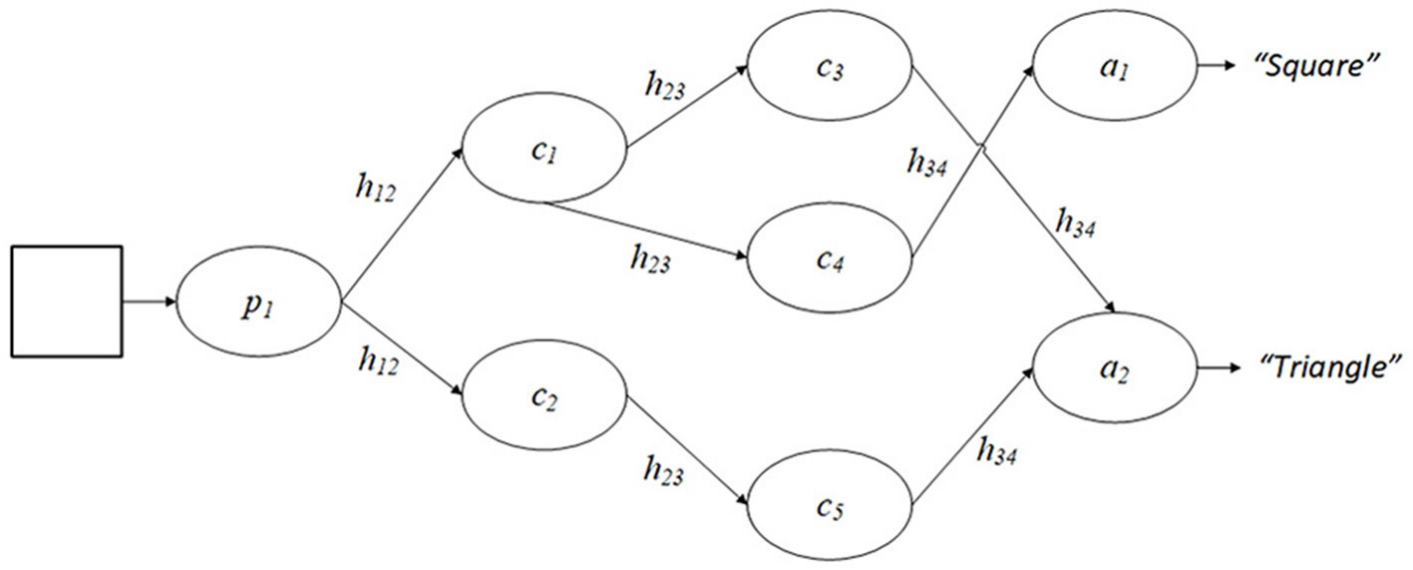
An example of an agent's clip space in a PS framework. Here, a percept clip *p*_1_ is triggered by a shape in the agent's environment and, under the traditional PS design, the agent will take a random walk through the clip space and, based on the *h* values present, will reach one of two action clips, each of which provide a different answer.

With this design, the behavior of PS agents can be likened to mapping some input data *s* to an action *a*. Figure 1 illustrates this design, using the description of shapes as an example. How an agent transitions between a percept and an actuator is determined by the conditional probability *p*^(*t*)^(*a*|*s*) at a time *t*. Under reinforcement learning, this conditional probability can be influenced by adjusting the edge weights or *h*-values of connections in the agent's clip space based on feedback from the environment:


(4) $$\begin{array}{l} h^{\left(t+1\right)}\left(c_{i},c_{j}\right)=h^{\left(t\right)}\left(c_{i},c_{j}\right)-\gamma \left(h^{\left(t\right)}\left(c_{i},c_{j}\right)-1\right)+\lambda \end{array}$$

In EPS and EEPS, this PS framework is applied in the simulation of matching-to-sample (MTS) tasks, a popular procedure of testing SE abilities via establishing several new baseline relations and subsequently evaluating if symmetry, transitivity, and equivalence relations form as a result of this training (Steele and Hayes, 1991). Participants are presented with a sample stimulus and two or more comparison stimuli, which can include letters, images, word, audio, etc., are commonly fabricated precisely for the experiment and are designed in most cases to be distinct from real-world stimuli. The participant selects one of the comparison stimuli, which, either correct or incorrect, helps to reinforce the baseline relationships between one or more pairs of stimuli. After reaching some criterion for correct matches (a mastery criterion), the experiment will often continue to train new relations and test the participant's formation of reflexivity, symmetry, transitivity, and equivalence relations, thus determining the participant's equivalence responding abilities.

The differential reinforcement of the MTS procedure integrates cleanly with the PS and reinforcement learning framework. With PS, agents in EPS and EEPS perceive sample and comparison stimuli in the environment as percepts and actions. A clip *c*_*s*_ ∈ *C* is created for each stimulus observed. Connections between the sample stimulus and comparison stimuli (*c*_*a*_) are initialized with a default edge weight value *h*_0_. If the agent matches the correct pairs of stimuli, the weight of the edge between the two stimuli in the agent's clip space is increased by 1. If incorrect, the edge weight is decreased by 1. These edge weights are used to calculate the transition probability between the sample stimulus and comparison stimuli, with both models utilizing a variant of the softmax formula:


(5) $$\begin{array}{l} p^{\left(t\right)}\left(c_{i}|c_{j}\right)=\frac{e^{\beta_{h}^{\left(t\right)}\left(c_{i},c_{j}\right)}}{\sum_{k}e^{\beta_{h}^{\left(t\right)}\left(c_{i},c_{k}\right)}} \end{array}$$

Here, the β_*h*_ variable is utilized to control the speed of learning, with smaller values of β_*h*_ resulting in the agent learning new relations slower, and also being less likely to form transitivity relations. Mofrad et al. demonstrate each model via a series of experiments which illustrate, with EPS, the framework's ability to replicate and extend classical experiments, and with EEPS, how the agent and environmental parameters affect the behavior of agents and the simulation.

A significant difference between EPS and EEPS is the mechanisms utilized in establishing derived relations during and after training. EPS possesses a *ad-hoc* policy that assumes the formation of transitivity and equivalence relations in training but only calculates their weights during testing, hence the model prevents the agent's memory from changing after completing training. The method for calculating these derived relations varies, with the authors testing random walks, max product, and memory sharpness algorithms as approaches. Meanwhile, EEPS creates transitive and equivalence relations via an approach that is akin to an “offline replay” system—rather than calculating when required, the agent's entire network is updated using a modified variant of a de-noising algorithm called Network Enhancement (NE), which is applied once the agent has completed the training phase and before evaluation. Designed by Wang et al. (2018), NE aims to provide “a better representation of the underlying module membership” of nodes in a network vs. its original structure. This involves creating a localized network T within the same set of nodes and obtaining K-nearest neighbors for *i*th node, denoted $N_{i}$, in a two step process:


(6) $$\begin{array}{l} P_{i,j}\leftarrow \frac{W_{i,j}}{\sum_{k\in N_{i}}W_{i,k}}{𝕀}_{\left\{j\in N_{i}\right\}},\;T_{i,j}\leftarrow \overset{n}{\underset{k=1}{\sum }}\frac{P_{i,k}P_{j,k}}{\overset{n}{\underset{v=1}{\sum }}P_{v,k}} \end{array}$$

The first step calculates a transition probability matrix labeled *P* using an indicator function 𝕀. The second step computes the localized transition matrix T using this transition matrix. This approach measures local affinity and prioritizes connections between nodes “within three orders of distance” away. EEPS contains two different versions of NE: the original version proposed by Wang et al. (called Symmetric Network Enhancement or SNE), which applied the localized network T in the diffusion process:


(7) $$\begin{array}{l} W_{t+1}=\alpha T\times W_{t}\times T+\left(1-\alpha \right)T \end{array}$$

and a modified version which replaces the localized network with a transition probability matrix called directed network enhancement (DNE) which the authors state “provides a better formation of classes.” This version substitutes the localized network for the transition probability matrix *P*:


(8) $$\begin{array}{l} W_{t+1}=\alpha P\times W_{t}\times P+\left(1-\alpha \right)P \end{array}$$

The PS framework provides a transparent and understandable foundation for empirically examining linguistic relational networks, which is typically absent in contemporary models built on opaque AI designs that have been criticized for inaccurate learning mechanisms and “biological implausibility” (Stork, 1989; Castro and Siew, 2020). Additionally, the clip space of agents in PS is akin to Tolman (1948)'s pivotal cognitive map design, which lends the model a reliable and supported internal representation. While either EPS or EEPS would be suitable for modification, the motivation behind utilizing Affinity is threefold. Firstly, the model's inclusion of the NE algorithm presented an opportunity to explore how the algorithm enables the formation of transitivity and equivalence relations. Secondly, inspired by discussions proposed in Mofrad et al. (2021), adjustments can be made to EEPS's source code to support the application of NE during the training stage, each time the agent achieves the mastery criterion. This alternative design can be contrasted against the original, and the effects of the algorithm on the agent's clip space can be visualized. Thirdly, a novel analysis can be carried out on how each design affects the higher-order properties of the agent's memory, as proposed in RDT (Belisle and Dixon, 2020b).

RDT merges SE and RFT with another account called Behavioral Momentum Theory (Nevin and Shahan, 2011), a quantitative approach to analyzing the effects of behavioral reinforcement by applying Newtonian mechanics. RDT adopts the same mathematical framework of Newtonian mechanics to describe behavioral networks, treating density, volume, and mass as abstract analogs of physical quantities. Under this design, a change in relational responding (Δ*R*) can be modeled as a counterforce −*x* against the relational mass of the network *Rm*:


(9) $$\begin{array}{l} \Delta R=\frac{-x}{Rm} \end{array}$$

This relational mass, which describes a network's resistance to change via counterconditioning, is equivalent to the product of the network's relational density *Rp* (the overall strength of relations within the network) and its relational volume *Rv* (the number of relations/stimuli within the network):


(10) $$\begin{array}{l} Rm=Rp\times Rv \end{array}$$

Relational volume and density are inversely related properties that allow RDT to predict non-linearity in equivalence responding. Belisle and Dixon also posit that relational networks high in volume and density are “highly resistant” to counterconditioning. The former is based on findings by Spencer and Chase (1996) and highlights that, in equivalence experiments, equivalence classes are not equal and “instead differ across several interactive dimensions.” Dixon et al. (2006) supports the latter, identifying relational resistance in perceived stimuli associated with firmly held beliefs.

As part of the overall RDT research programme, Belisle and colleagues have examined how networks with greater relational mass have acceleration and gravity, which affects other networks and the rate at which new stimuli are assimilated into the network (Belisle and Dixon, 2020a; Belisle and Clayton, 2021). Research has also been conducted to identify suitable measures for relational volume and relational density, with Cotter and Stewart (2023) testing nodal distance vs class size as measures for relational volume in MTS experiments. Utilizing four equivalence classes with a linear training structure (A-B-C-D-E), relational density was recorded using the participants' response latency (how quickly a comparison stimulus was selected). The authors noted that the nodal distance of two stimuli [defined as “the number of nodes that link two stimuli not related by direct training,” where a node is “a member of a equivalence class that has been directly trained to at least two other stimuli” (Fields et al., 1984)] was inversely proportional to relational density and thus is a better fit for relational volume than the class size.

This study and its findings highlight an opportunity to explore the simulation of RDT, identifying which metrics best capture density and volume in EEPS. By leveraging agents' “episodic and compositional memory” in EEPS, we can identify and analyse key indicators of these higher-order properties. Additionally, we can create an interface for EEPS that streamlines its operation and visualizes the internal clip spaces of agents, elucidating the iterative development of an agent's clip space at regular intervals and providing an additional level of interpretability upon the original design. This interpretability helps improve our ability to simulate equivalence responding, and therefore also works toward a better understanding of its emergence in human and animal populations.

In this paper, we present Affinity, an extension for EEPS that provides real-time visualizations of agent behavior. Built using the PyQT package, Affinity has a graphical interface where users can set model parameters, run multiple iterations of EEPS side-by-side, access and save visualizations and results, and directly modify relations in the agent's clip space mid-simulation. In the following sections, we will provide a detailed description of Affinity and its features, give a methodology for demonstrating Affinity, its RDT features, and the effects of NE in EEPS by recreating Cotter and Stewart (2023), outline the results of this demo, and discuss the implications for EEPS and future work on RDT, and future work on Affinity and computational modeling in Contextual Behavioral Science.

## 2 Method

Affinity is implemented in Python, which provides an accessible foundation for integrating it with various Python packages and EEPS. The source code is available on GitHub under an MIT License: https://github.com/jamosully/EEPS-Visualizations. An overview of the user interface, changes to the original EEPS code, visualizations of the agent's clip space, RDT metrics, and the procedure for the demonstration are provided in the subsections below. Further documentation on installation and use is provided alongside the source code on the GitHub repository.

### 2.1 Implementation of user interface

**Figure 2:**
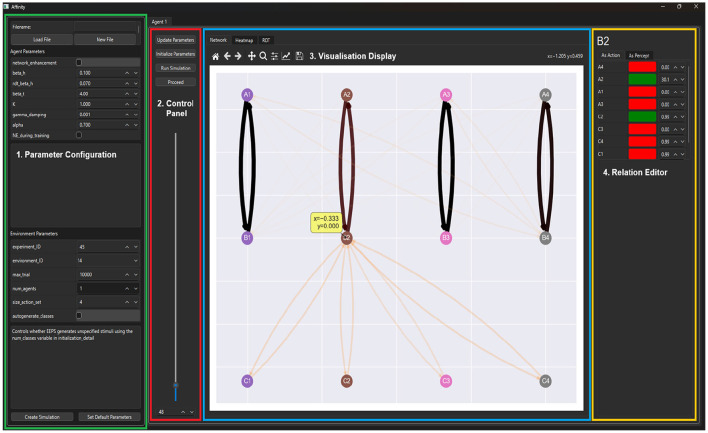
The GUI of Affinity consists of four main components. (**1**) The Parameter Configuration menu (green) allows users to adjust the settings of the EEPS model and load previous experimental configurations. (**2**) The Control Panel (red) provides interactions to control the progression of the experiment. (**3**) Visualization Display (blue) contains the graph, heatmap, and RDT visualizations, as well as the results once a simulation has concluded. (**4**) The Relation Editor (yellow) displays tables with the edge weight values of incoming and outgoing edges of a selected stimulus.

The interface of Affinity is built using PyQT (Riverbank Computing, 2012), an extensive library with a flexible modular design. Visualizations are integrated into the interface via packages such as matplotlib and networkx. Figure 2 displays the interface of Affinity, which consists of four components:

- Parameter configuration: on the left side of the interface, a menu is provided for selecting and altering model parameters. A description accompanies each parameter. Users can also load in results from prior experiments and set their current parameters as the default for subsequent launches. Affinity uses a JSON file to save each parameter's name, value, description, and widget type to alter the value (e.g., drop-down, toggle button, spin box, text field). This JSON file updates the initialization script from the original EEPS design.
- Control panel: the control panel contains a slider and spin box for selecting the step value that controls the rate at which visualizations are produced, with steps equal to the number of trials between visualizations. The four buttons above the slider are used to initialize the EEPS model, update its parameters, launch the simulation, and progress through it.
- Visualization display: to the right of the control panel is the visualization display, comprised of tabs that display the agent's clip space as a graph network and heatmap, as well as the RDT metrics and, upon completion of the simulation, the results of the experiment.
- Relation editor: to the right of the visualization display, the relation editor is where users can modify the strength of edges within the agent's clip space. By clicking on clips within the graph network visualization, users are provided with a breakdown of each clip's ingoing and outgoing relations, the weight of each relation, and whether the relation is part of the class. Users can alter the edge weight values presented, and upon progression, these modifications are made to the agent's clip space.

Affinity connects directly to the EEPS model, leveraging PyQT's multithreading, mutex, and event handling modules (Driscoll and Driscoll, 2018; Harwani, 2018) to create a custom simulator object each time the user launches a simulation. This design allows Affinity to separate the front-end GUI from the EEPS process, improving performance. Additionally, in combination with the tabular design of the main interface, Affinity can run multiple versions of EEPS simultaneously.

### 2.2 Modifications to EEPS

Developing Affinity and simulating, Cotter and Stewart (2023) required several modifications to EEPS, the primary of which was implementing a second version of NE that could be applied during the training phase of a simulation. The algorithm's design followed the same steps as the original version, but rather than returning a matrix of transition probabilities, this new version returns a de-noised version of the agent's clip space with adjusted edge weights each time the agent reaches the mastery criterion for the training phase. Additionally, as NE's purpose is to introduce derived relations in the agent's network, baseline relations are not included in the application of NE. Other minor modifications include changes to the experimental loop, which initiates the agent's training process, which is now expanded to pause the simulation when Affinity's step value has been reached and create visualizations, and the EEPS environment now keeps track of the success rate of each class during an experiment, which is used as one of three potential measures of relational density.

### 2.3 Agent network visualization

Visualization of the agent's clip network is displayed in two different formats. The first is a graph network visualization inspired by both the figures present in Briegel and De las Cuevas (2012) and the visualizations provided in the appendix of Mofrad et al. (2020). In the latter, the agent's clip space is represented by each stimulus of a class aligned with the others in the same column. This orientation is interchangeable between experimental setups and can be scaled up upon introducing additional classes and stimulus types. Implementation of this design was also assisted by EEPS using the networkx library to store an agent's memory during simulation. Networkx provides built-in support for drawing networks via the matplotlib library (Hagberg et al., 2008).

**Figure 3:**
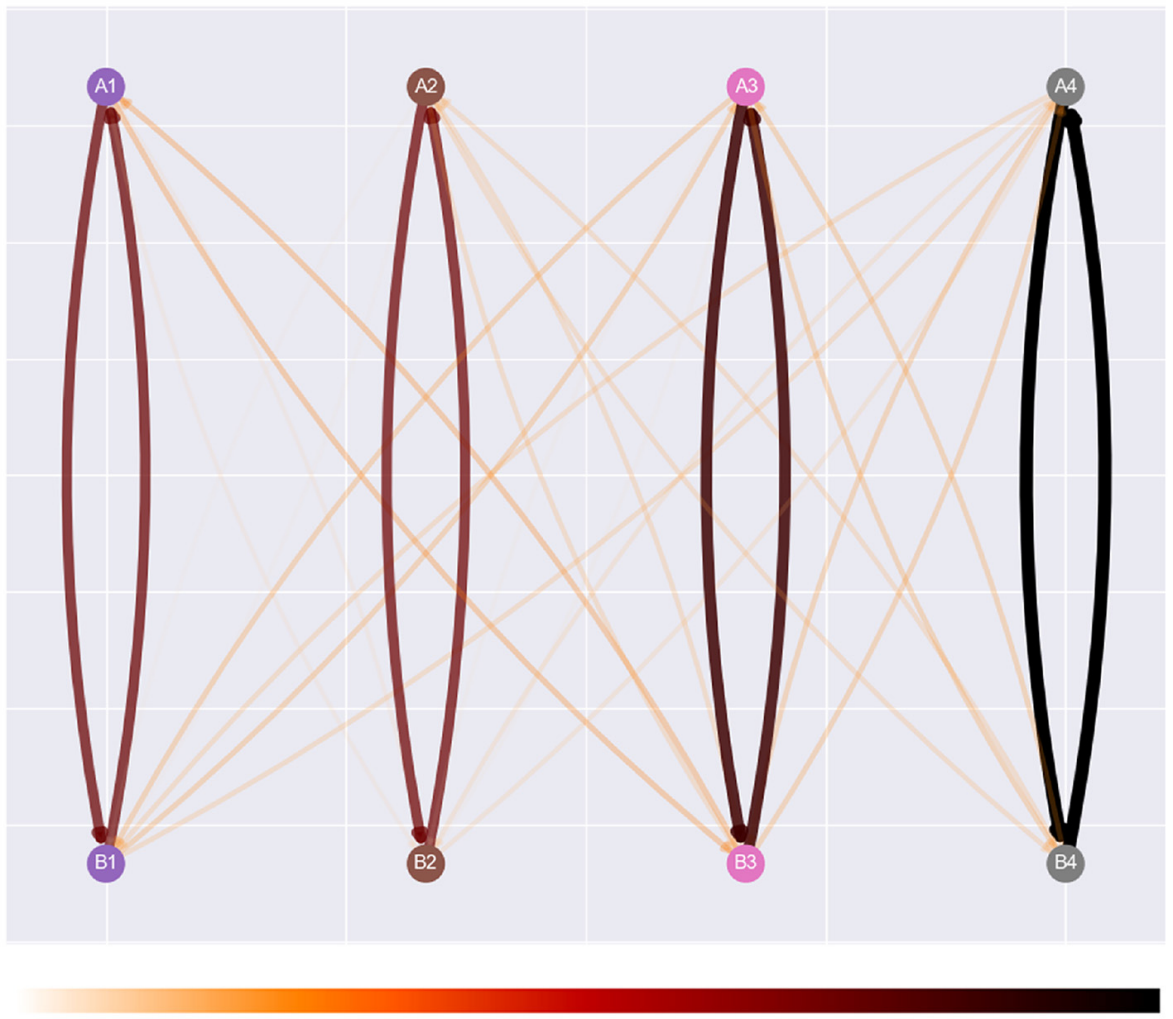
Affinity's graph network visualization, with its color map, “gist_heat_r.” This is a reversed version of the original color map, which visualizes stronger relations in darker colors and weaker relations with lighter colors.

Figure 3 illustrates the visualization design. The rationale behind the visuals focuses on interpretability in the interface and on paper. The design of the graph and edges adopts heuristics outlined by Bennett et al. (2007), and this consideration has also been considered when visualizing the strength of each edge weight. The color map presented at the bottom of Figure 3 utilizes darker and thicker edges to illustrate stronger relations, with the white edges visible against the gray background of the graph. The opacity of edges is calculated based on the normalized weights across the entire network, but is clamped at 0.33 to prevent them from becoming too transparent. More substantial edges also possess thicker edges than their weaker counterparts. Overall, the combination of these features provides sufficient clarity regarding the strength and structure of the agent's clip space.

**Figure 4:**
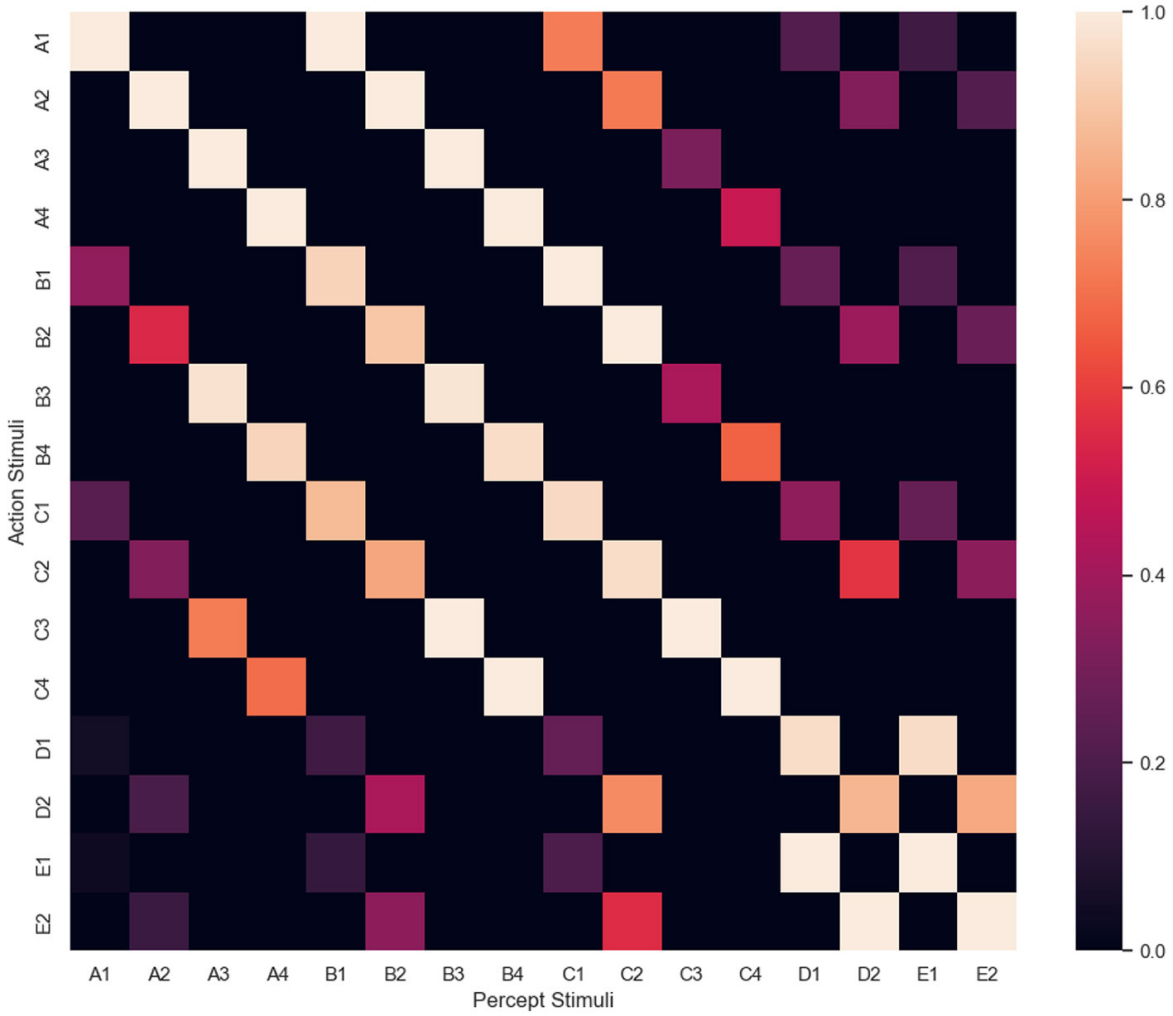
The heatmap visualization, with percept/sample stimuli along the *x*-axis, and action/comparison stimuli along the *y*-axis. This heatmap was obtained at the end of an experiment, where NE created several transitive and equivalence relations.

While developing Affinity, we noted that the graph network visualizations could present issues. For example, as the number of stimuli in the network increases during an experiment, the number of relations can increase significantly, especially once NE has been applied. This can result in a cluttered visualization, potentially affecting user comprehension (Glazer, 2011). Therefore, a heatmap visualization is also displayed using the seaborn package (Waskom, 2021). Illustrated in Figure 4, the visualization provides a suitable alternative to the graph network visualization and captures the multivariate data from the agent's clip space. By applying a normalized color space, attention can be drawn to specific value ranges central to the data being visualized (Ward et al., 2010). In Affinity, the heatmap visualizes a normalized matrix of the edge weights, with percept/sample stimuli along the *x*-axis, and action/comparison stimuli along the *y*-axis. Figure 4 represents a heatmap at the end of training, once NE has been applied. Hence, transitive and equivalence relations are present.

### 2.4 Relational density theory metrics

**Figure 5:**
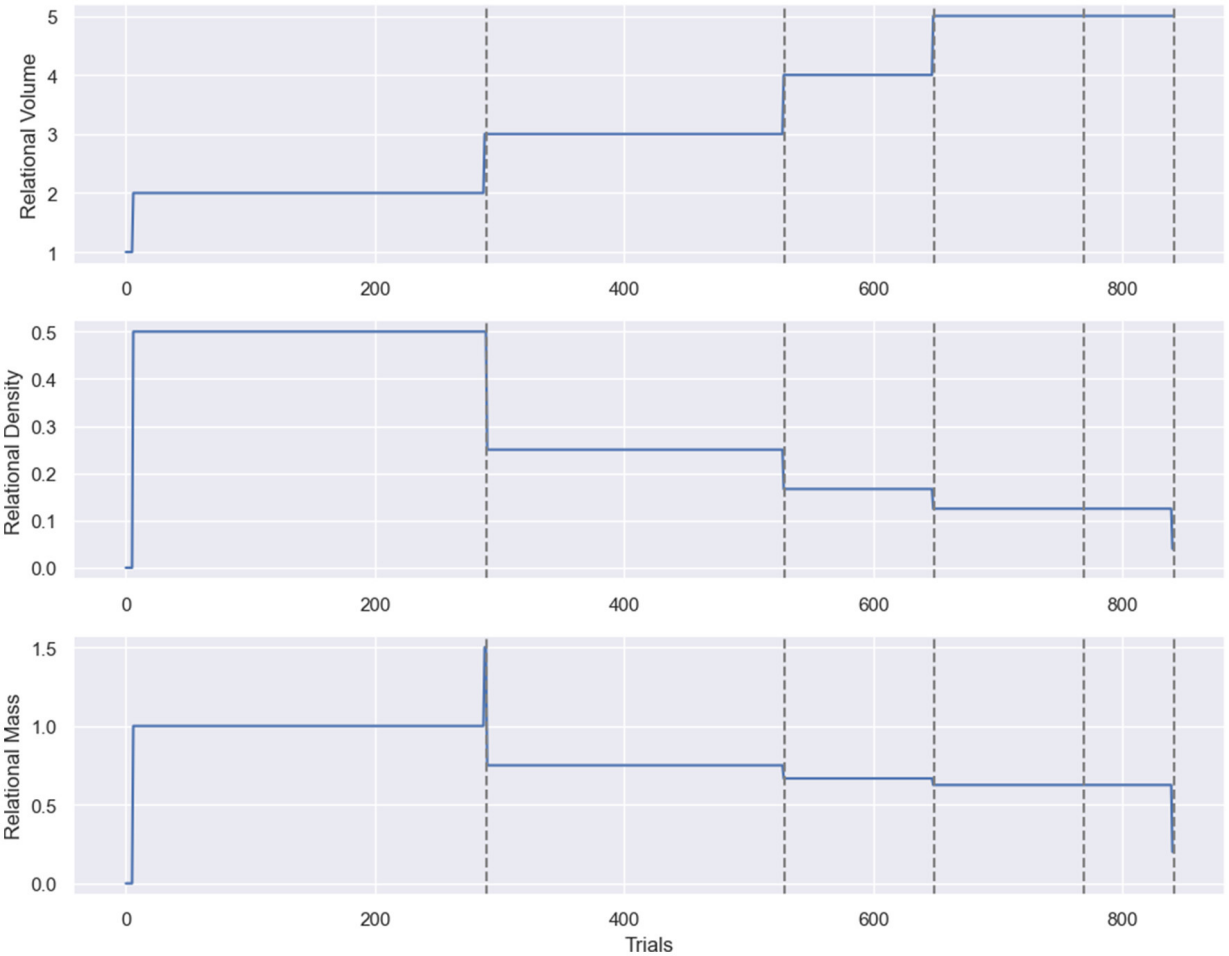
The RDT metric visualization provided by Affinity. From **(top to bottom)**: relational volume, density, and mass. The *x*-axis represents the number of trials/steps in the simulation, and the dashed lines represent the transitions between training phases. In the example above, the relational volume is the true nodal distance, and the relational density is the mean transition probability. The notable spike in the relational density measure results from the selected class only having a single stimulus for the first few trials of the simulation.

Figure 5 illustrates the RDT tracking in Affinity, with three plots for each class of stimuli, detailing the step-by-step changes to relational density, volume and mass within the agent's clip space. Each metric is displayed as a continuous line plot, showing the entire breadth of data obtained during a simulation. Two drop-down boxes in the main display allow the user to switch between which of the relational volume or density measures is being visualized. Changing this version also updates the relational mass figure. Alongside these options are a set of buttons, one for each class, that can be clicked on to switch between the class being visualized.

With access to an agent's internal clip space, we can explore more measures for relational density and volume beyond those present in Cotter and Stewart (2023). These are the measures of relational volume available in Affinity.

- Nodal distance: in Affinity, nodal distance comes in two forms. The first is based on Cotter and Stewart (2023)'s definition of nodal distance as the number of nodes [where a node is a stimulus with at least two trained relations (Fields et al., 1984)] in the agent's memory that link two stimuli that have not been directly trained. This is labeled as *empirical nodal distance*. The other form of nodal distance is *true nodal distance*, which records nodal distance as the shortest path between any stimuli in a class (regardless of the number of trained relations), including any transitivity/equivalence relations formed throughout training. The transparent memory of agents in EEPS affords this form of nodal distance, which is more susceptible to fluctuations from differences in training structure and NE. For both measures, the total of each class is recorded.
- Class size: this is the other measure of relational volume presented in Cotter and Stewart (2023)'s study and is the number of stimuli in each class. While Belisle and Dixon (2020b) define relational volume as the number of relations in a network, class size and empirical nodal distance are highlighted in Belisle and Dixon (2020a) as estimates of relational volume, especially in experiments where specific relations are not trained or tested. Therefore, the implementation of both in Affinity supports the comprehensive replication of studies on RDT in MTS tasks.
- Number of relations: this measure is suggested in Belisle and Dixon (2020a) as the relational volume of a network and can be directly correlated to class size. The significant difference between the two is that the number of relations will be directly affected by including the NE algorithm, increasing the number of relations with the formation of reflexivity, transitivity and equivalence relations.

We have also implemented the following measures of relational density in Affinity:

- Mean transition probability between intra-class stimuli: using the same softmax distribution function that an agent in EEPS uses to select comparison stimuli, this measure takes an average over the distribution of all the edges between stimuli within a class, including baseline, symmetry, and transitivity relations. While, in some cases, this measure can roughly equate to 1/number of stimuli, the measure is particularly useful when interacting with Affinity's relation editor and with various values of β_*h*_, which affects an agent's rate of learning.
- Class accuracy: this measure is the correct matches between sample and comparison stimuli for stimuli in a class, divided by the overall number of trials (baseline, symmetry, transitivity) within that class. The accuracy of matching in MTS procedures has been used in prior studies as a measure of performance (Saunders et al., 2005), and it is testable in both EEPS agents and human participants. Additionally, as with class size and nodal distance for relational volume, class accuracy is readily identifiable measure in real-world experiments, and therefore can potentially serve as a estimate for relational density.
- Mean *h*-value/edge weight: using the weight of edges within the agent's clip space, this measure can capture the strength of all relations between stimuli within a class. Alongside the mean transition probability, this metric leverages the structure of the agent's clip space, with the main difference being that *h*-values in the agent's clip space are unbounded and, therefore, the mean *h*-value can be skewed by outlier relations that have received significantly more training than their counterparts. However, unlike the mean softmax probability, the mean edge weight is not affected by β_*h*_, and rather parameters such as *K*, γ, and α.

Alongside the visualizations created during the runtime of a simulation, Affinity also provides additional visualizations at the end of a simulation. Alongside the results in the original version of EEPS, Affinity provides line graphs showing the change in each measure of relational density and volume across all classes and line graphs for each type of relational mass. Another line graph depicts all relational mass types together, and a boxplot displays Pearson's correlation coefficients for all relational mass combinations. These coefficients help analyse which measures best capture the inverse relationship between relational volume and density.

### 2.5 Experiment design

To demonstrate Affinity and its features, we aimed to replicate a modern study that utilized the MTS procedure and involved RDT. Highlighted in Section 1, Cotter and Stewart (2023) has investigated the role of volume in RDT using MTS-based experiments, finding that nodal distance shares a stronger inverse relationship with relational density than class size. Therefore, this study is an ideal candidate for demonstrating Affinity's effectiveness as a tool for experimental analysis. Additionally, while the author's use of response latency as relational density could not be extended to EEPS, exploring the potential alternatives outlined in Section 2.4 is a unique opportunity and has the potential to inspire further real-world studies as well.

The design of the MTS experiment in Cotter and Stewart (2023) is as follows. Four classes were utilized, with classes 1 and 2 containing five members and classes 3 and 4 containing three. Four stimuli (D3, D4, E3, E4) were utilized as comparison stimuli but not directly trained. Phase 1 trained A–B relations and Phase 2 trained B–C relations for all four classes, while Phase 3 trained C–D and Phase 4 trained D–E relations for classes 1 and 2. Phase 5 conducted mixed training of all relations across all four classes and tested entailment relations (A–C, C–A, C–B, B–A for all classes, D–C, E–D, A–E, E–A for classes 1 and 2). Phase 6 performed counterconditioning on A–B relations in all four classes, with new relations A1–B2, A2–B3, A3–B4, and A4–B1. Prior correct responses now resulted in negative feedback. The final phase, Phase 7, retested all trained and tested relations in Phase 5. The authors used this design to explore several predictions, including two on relational density, and how the class size/nodal distance affected the strength of relations. Firstly, relations within large classes were predicted to be less dense. Contrary to the original prediction that larger classes would exhibit lower density, Cotter and Stewart found that smaller classes (three members) were less dense in more than half the cases. Secondly, relations with a larger nodal distance between their relata would be less dense. The results showed that relations with a nodal distance of 3 were less dense than those with a nodal distance of 1 in 76% of cases.

To demonstrate Affinity, we utilized a recreation of the first five phases of this study as a platform to explore the software's new features and the initial insights they provide. This demo will explore how the simulation results align with the first two predictions and their respective findings outlined in Cotter and Stewart (2023). We can also take a step further by tracking Pearson Correlation Coefficients for each version of relational mass created as a product of the seven measures of relational density and mass available in Affinity. These findings will allow us to examine which factors best capture the inverse relationship posited by Belisle and Dixon (2020b). We will also recreate the study under two conditions in the two environments. The first condition is based on the original design of EEPS, with NE being carried out on the agent's clip space at the end of training. The second condition will apply NE once the agent reaches the mastery criterion for each training phase. With these conditions, we will provide a clearer understanding of the effect of NE on the agent's network, the predictions made by Cotter and Stewart, and the RDT tracking of Affinity. Overall, this simulation will serve as a proof-of-concept of Affinity: outlining one of many potential use cases and highlighting the novel features that set it apart from EEPS.

With these objectives outlined, these are the parameters of our demonstration: γ = 0.001, *K* = 1, β_*h*_ = 0.1, β_*t*_ = 4, and α = 0.7. These are according to the default parameters outlined in experiment 1 of Mofrad et al. (2021). The mean transition probability measure of relational density will also be calculated using β_*h*_. For each version of the simulation run, we will utilize 15 agents, the number of participants that completed phase 7 in Cotter and Stewart's study, increasing the accuracy of our results. Each agent must achieve a 0.9 mastery before progressing to the next training stage. We will utilize DNE instead of SNE for all applications of NE, based on Mofrad et al.'s testimony that SNE does not entirely control the formation of symmetry and transitivity relations.

## 3 Results

**Figure 6:**
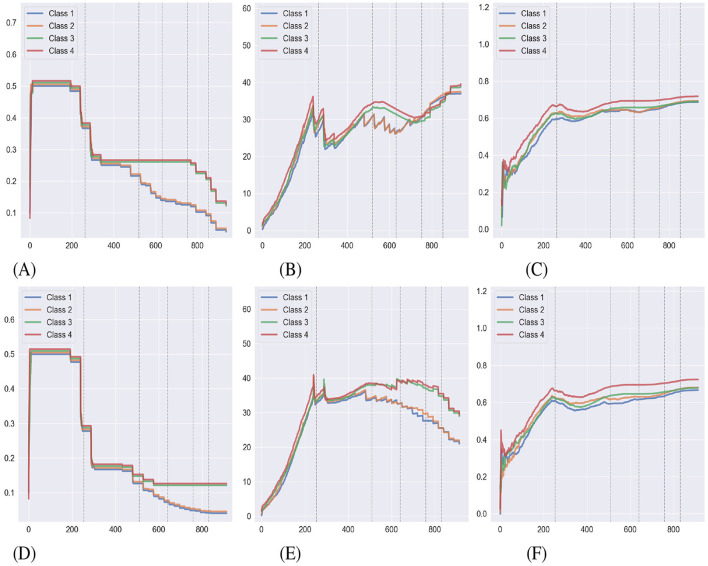
The average behavior of relational density metrics during the Cotter and Stewart study simulations under both NE conditions. **(A**, **B**, **C)** Shows measures during a simulation with NE carried out at the end of training. **(D**, **E**, **F)** Illustrate the same under a simulation where NE has been applied during training (lines have been offset for visibility). The dashed gray lines indicate transitions between the training phases. **(A)** Mean transition probability. **(B)** Mean edge weight. **(C)** Class accuracy. **(D)** Mean transition probability. **(E)** Mean edge weight. **(F)** Class accuracy.

Table 1 displays the mean performance of the 15 agents in both conditions, indicating little difference in time and mastery. Figure 6 displays the performance of each relational density measure under both NE conditions as a mean of 15 agents completing the experiment. The Figures 6A–C detail the behavior of each measure in a simulation where NE is only carried out at the end of the study. With both the mean transition probability and mean edge weight, classes 1 and 2 have a lower relational density during the latter stages of the simulation as D1, D2, E1, and E2 are introduced. This result loosely supports the two predictions in Cotter and Stewart regarding relational density, but doesn't align with the findings. The Figures 6D–F show the same measures for a simulation where NE is applied during training. As with the prior condition, the results for mean transition probability and mean edge weight are aligned with Cotter and Stewart's predictions but not their findings. This condition has also had a noticeable effect on the mean transition probability, providing a significantly lower value of relational density in the second half of the experiment than when compared with their no-NE counterparts, likely as a result of NE adding additional edges to the agent's clip space with weak *h*-values/edge weights. This effect can also be observed in the mean edge weight decreasing toward the end of the simulation. The class accuracy metric follows a similar trend in both conditions, stabilizing to ~0.7 during the simulation, and does not follow a similar, decreasing trend as does its counterparts.

**Table 1:** Table containing the results of each training phase over the 15 simulations.

**Training**	**Number of trials**	**Average time**		**Average mastery**	
		**No NE during training**	**NE during training**	**No NE during training**	**NE during training**
Phase 1: AB (A1B1, A2B2, A3B3, A4B4)	48	5.466	5.267	0.963	0.942
Phase 2: BC (B1C1, B2C2, B3C3, B4C4)	48	5.333	5.333	0.947	0.949
Phase 3: CD (C1D1, C2D2)	24	4.666	5.400	0.950	0.967
Phase 4: DE (D1E1, D2E2)	24	5.000	4.933	0.944	0.958
Phase 5: Mixed (AB, BC, CD, DE)	72	1.400	1.000	0.943	0.996

Time here represents the number of times that particular phases had to be repeated to achieve the 0.9 mastery criterion. Under both conditions, agents completed each phase of training in a comparable number of repeats.

**Figure 7:**
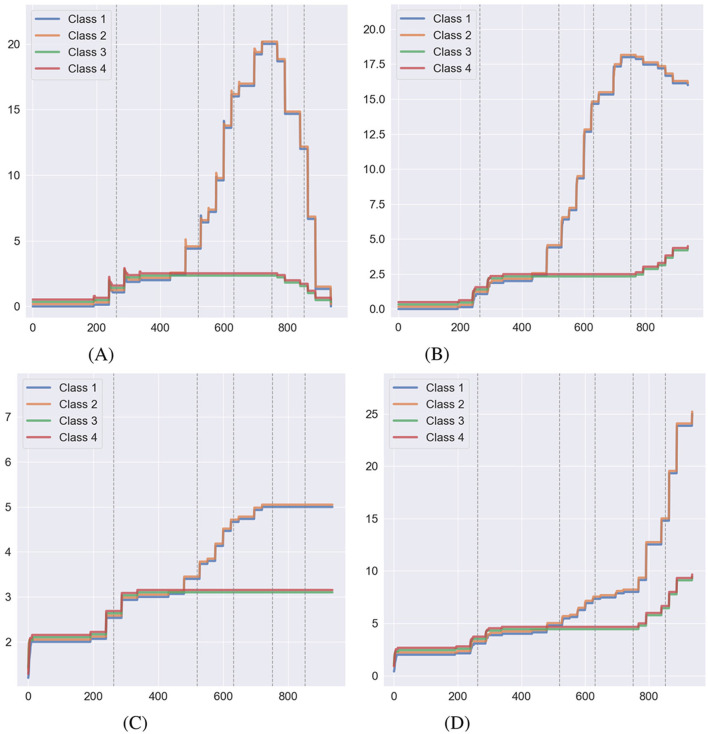
Behavior of relational volume measures during a simulation of Cotter and Stewart where no NE was carried out during the training process, taken as an average over 15 simulations (lines have been offset for visibility). The dashed gray lines indicate transitions between the training phases. **(A)** True nodal distance. **(B)** Empirical nodal distance. **(C)** Class size. **(D)** Number of relations.

**Figure 8:**
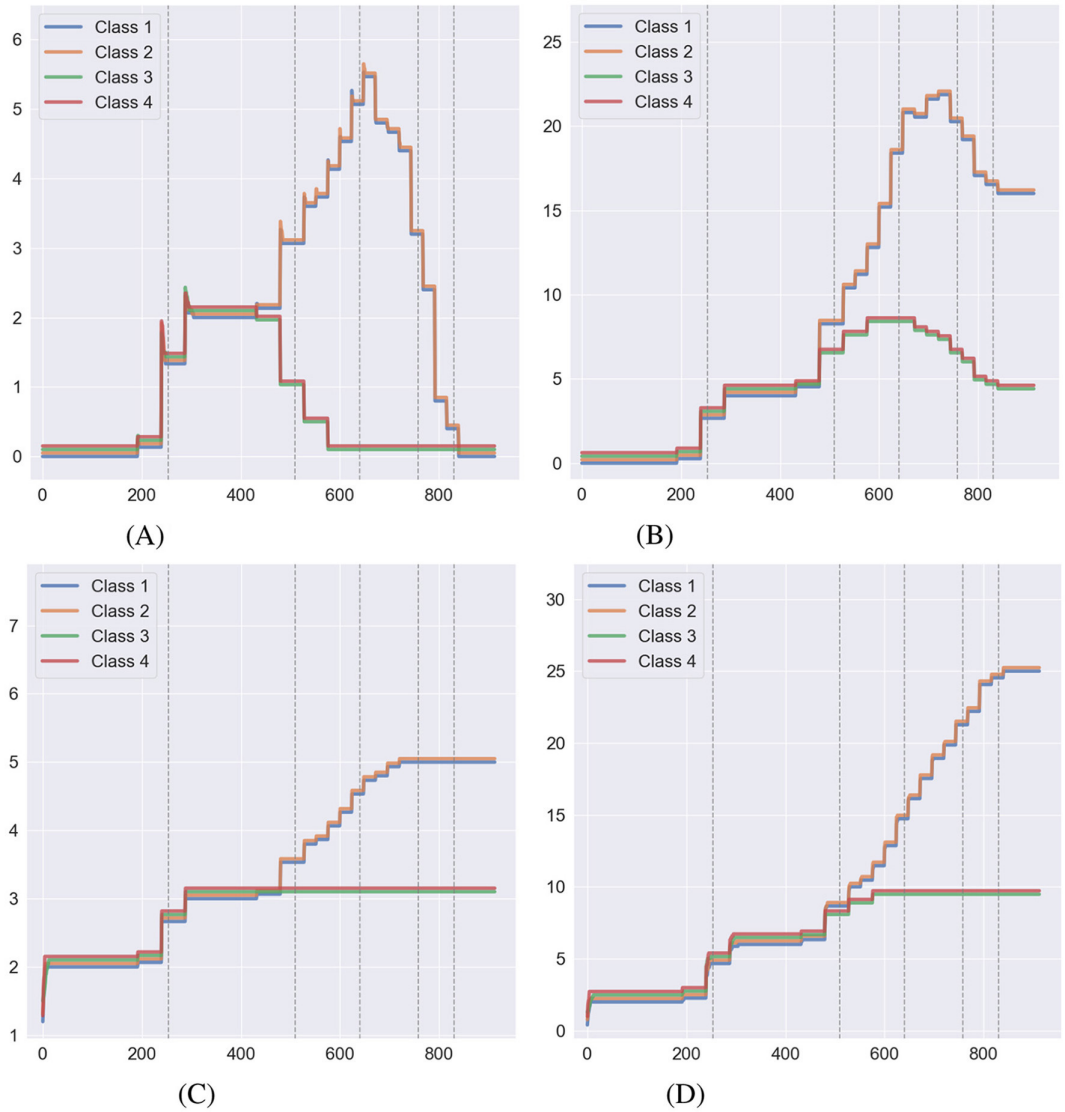
Behavior of relational volume measures during a simulation of Cotter and Stewart where NE was carried out during the training process, taken as an average over 15 simulations (lines have been offset for visibility). The dashed gray lines indicate transitions between the training phases. **(A)** True nodal distance. **(B)** Empirical nodal distance. **(C)** Class size. **(D)** Number of relations.

Figure 7 shows the behavior of relational volume measures without NE during training, while Figure 8 illustrates the same with NE applied during training. Figures 7A, B, 8A, B display the two forms of nodal distance in Affinity: true nodal distance and empirical nodal distance. As outlined in Section 2.4, the true nodal distance utilizes transitive and equivalence relations in its calculation. It does not conform to the exact definition of a node as the empirical nodal distance, hence why the true nodal distance is greater during the second half of the experiment. As with relational density, NE has affected measures in both conditions. When NE is carried out at the end of training, true nodal distance decreases as the number of direct connections between relations increases. Meanwhile, the number of relations increases with the alterations to the agent's clip space. As for the conditions where NE is carried out during the training stage, the formation of transitive and equivalence relations at each phase has curbed the true nodal distance measure, which decreases to around zero after the 800th step. The number of relations continually increases throughout the simulation. In both conditions, the class size measure remains stable.

**Figure 9:**
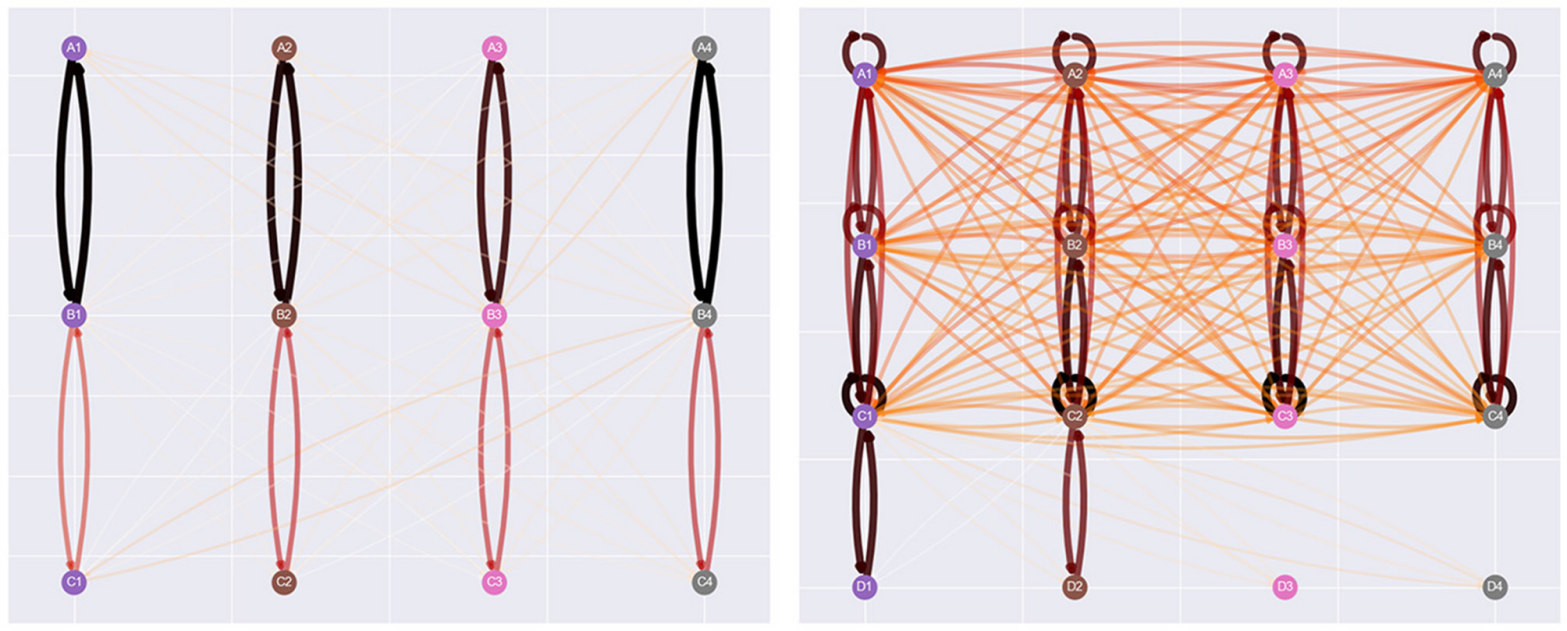
Network visualizations of the 500th step of the Cotter and Stewart simulation, with the no NE condition on the left, and the NE during training condition on the right. NE has created several additional relations, including inter-class relations between stimuli introduced in the same batch of sample stimuli and comparison.

Figure 9 provides a clearer picture of the effect of NE during training. Each visualization of the agent's network is taken at the 500th step of the simulation. In the left network, where NE hasn't been applied at this point, the only relations the agent has generated are the baseline, symmetry, and incorrect relations. Meanwhile, the visualization on the right indicates the effect of NE during training. The agent has generated numerous additional relations, including reflexive and inter-class relations between stimuli introduced within the same phase as comparison stimuli. This alteration of the agent's clip space explains several of the differences between conditions provided in Figures 6–8.

**Figure 10:**
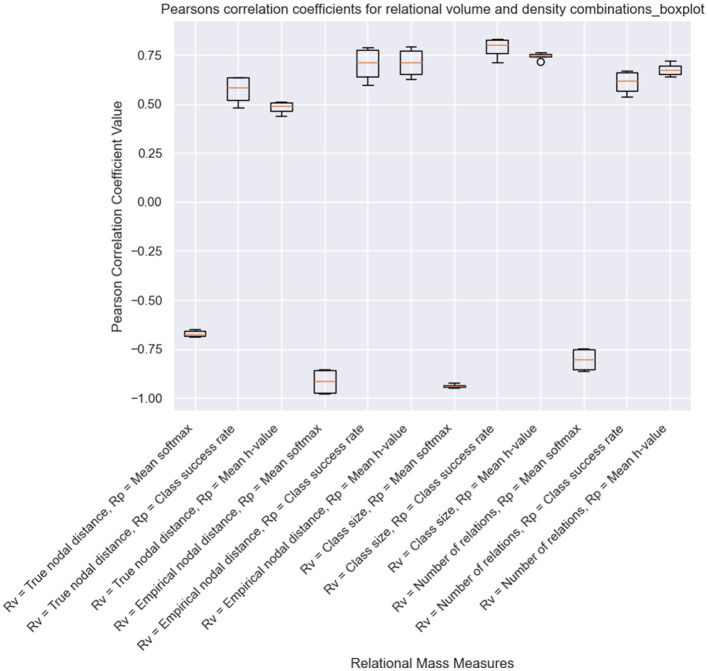
Pearson correlation coefficients for a simulation of Cotter and Stewart's study with NE only performed at the end of the experiment.

The final results we have obtained as part of this demonstration are the correlation coefficients for each version of relational mass. As shown in Figure 10, for the simulations where NE is only carried out at the end of the training, the mean transition probability combined with several of the measures of relational volume (mean coefficients with: true nodal distance = −0.671, empirical nodal distance = −0.915, class size = −0.937, number of relations = −0.803), best captures the inversely proportional relationship that Belisle and Dixon (2020b) proposed. Contrary to Cotter and Stewart (2023), class size was a better measure of relational volume than true and empirical nodal distance in this condition. This is likely a result of changes in the training order across the fifteen simulations (i.e., which stimuli are presented first at each phase of the experiment), and the application of NE at the end of the experiment. Additionally, the empirical nodal distance is a better fit for relational volume than the true nodal distance, which, again, is affected by the training structure and NE at the end of training. The NE process, as highlighted in Figure 9, strengthens and creates new intra-class relations between stimuli with large empirical nodal distances (e.g., A1–D1, B1–E1), which in turn greatly reduces the true nodal distance of an entire class.

Meanwhile, when NE was included during the training process, the inter-quartile range of most measures has increased, indicating a greater variance in performance of each version of relational mass. This is likely a result of NE's effects on specific measures of relational volume and density, such as the true nodal distance, number of relations, and mean edge weight/*h*-value. The measures that utilize the mean transition probability as density remain as the best for capturing the inverse proportional relationship between relational volume and density (true nodal distance = −0.435, empirical nodal distance = −0.870, class size = −0.938, number of relations = −0.879). As with the other condition, when calculated with mean transition probability/softmax, class size has performed better than empirical nodal distance, and empirical nodal distance has performed better than true nodal distance. Also, there is an increase in the coefficient for mean softmax and true nodal distance, obviously as a result of NE introducing transitive and equivalence relations at earlier phases of training.

## 4 Discussion

We have presented Affinity, a novel visualization tool that illustrates agent behavior with visualizations and RDT-driven models of their internal behavior. We have provided an overview of the software, outlining its features and visualizations, followed by a demonstration with the recreation of a modern MTS study on RDT (Cotter and Stewart, 2023), examining potential measures of relational density and volume and the effect of EEPS's NE algorithm on the agent's development. These sections have helped to highlight Affinity's capability to provide novel and testable insights on RDT and computational modeling of SE.

**Figure 11:**
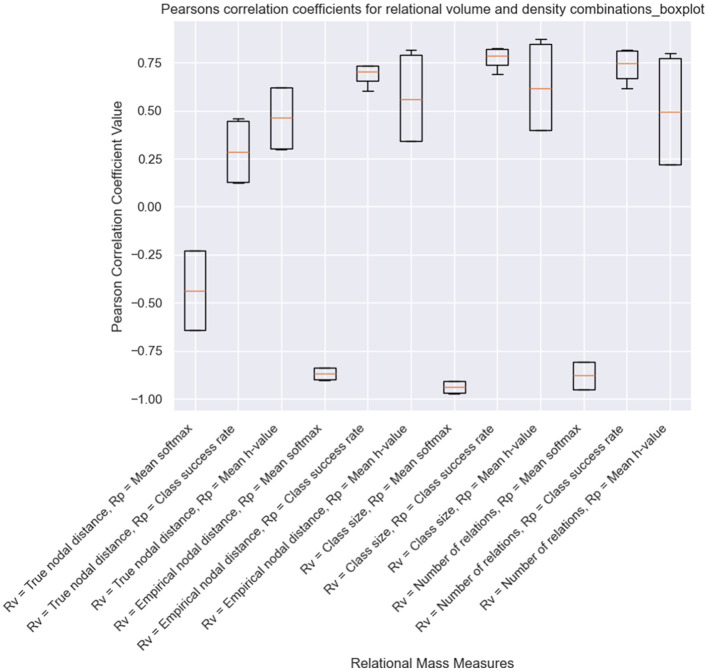
Pearson correlation coefficients for a simulation of Cotter and Stewart's study with NE performed during the training process.

While informal, our results show what insights can be obtained from Affinity, and the simulation of Cotter and Stewart (2023) has indicated the need for further research on RDT. The correlation coefficients presented in Figures 10, 11 indicate that, in EEPS, class size is a better measure of relational volume vs. either the true nodal distance or the empirical nodal distance of the agent's clip space. This is likely a result of differences in the training order across the fifteen simulations and the effects of NE altering the structural connectivity, either during training or at the end of the simulation. At the same time, Affinity, built upon EEPS's transparent design of the agent's memory, can obtain a more accurate measure of the relations between stimuli during the MTS procedure. With this access, Affinity discloses one possible version of the behavior of the fluctuating relational network that is constructed as part of the MTS procedure and illustrates the need for further research on RDT. Meanwhile, it appears that the mean transition probability of each class is the most suitable metric for relational density and a reliable alternative to response accuracy. In both conditions, the measure shared the strongest inverse correlation with the four measures of relational volume.

The ability to monitor two versions of nodal distance, true and empirical, has provided Affinity with an additional perspective on the behavior of individuals under the MTS protocol. While the empirical nodal distance captures a predictable and measurable metric solely from the training structure, the true nodal distance only exists thanks to the transparent design of agents in EEPS. Symmetry and transitivity probes would need to be carried out alongside baseline training for the true nodal distance to be measured in humans. An integrated training-and-testing structure has been used in classical studies such as Pilgrim and Galizio (1995).

**Figure 12:**
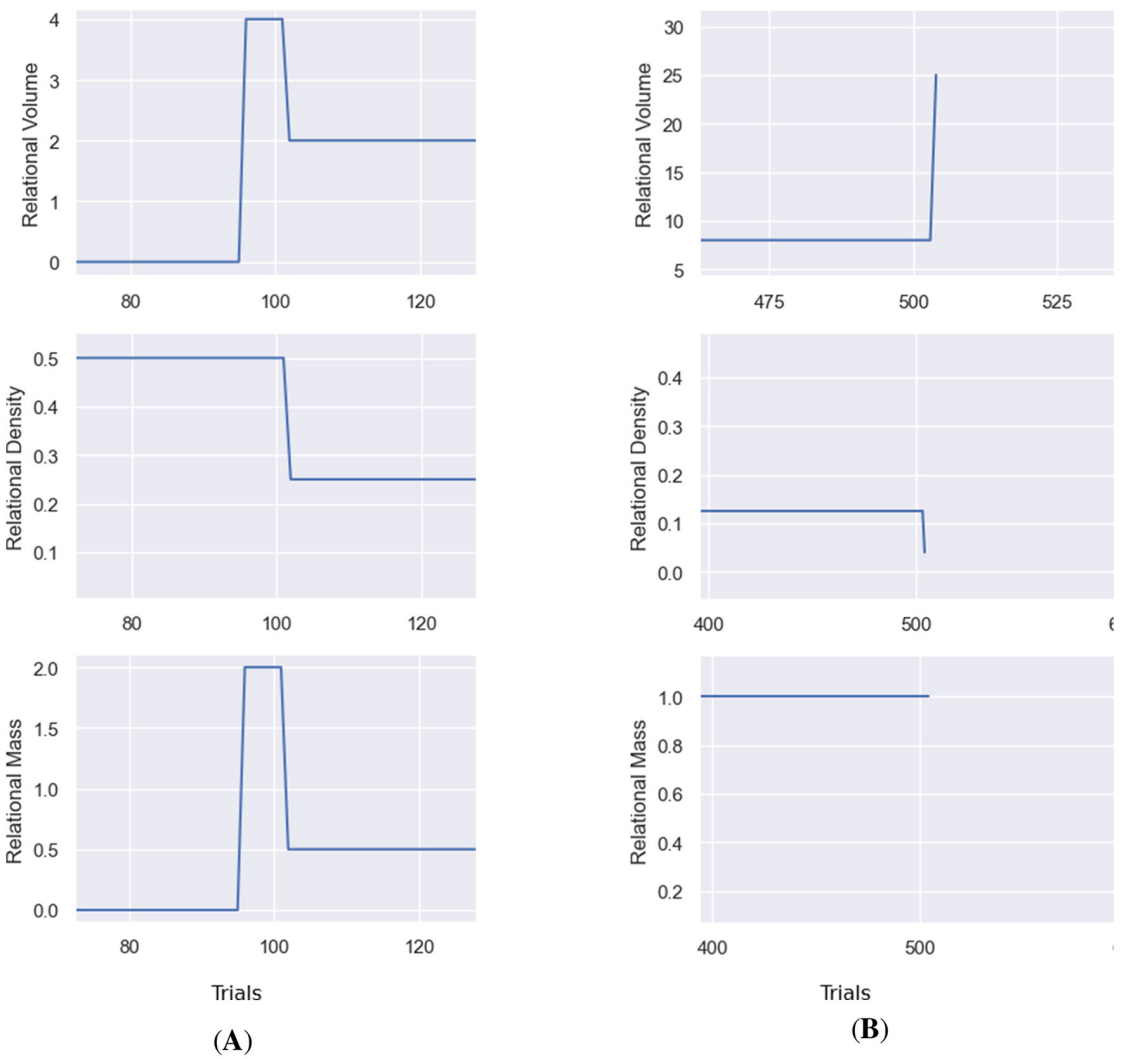
Examples of the spiking of measures of relational volume. **(A)** Shows a spike in the true nodal distance caused by introducing a stimulus as a comparison stimulus before direct training. **(B)** Illustrates a spike in the number of relations, following NE at the end of the training phase.

The behavior of the true nodal distance measure in Affinity highlights an unusual feature of the metric (at least in its implementation in Affinity). As demonstrated in Figure 12, during simulations, when new stimuli are introduced as comparison stimuli and are not directly trained to their corresponding class upon their first sighting, the actual nodal distance between the comparison stimuli and its sample stimuli is, for a few steps, substantial as a result of the shortest distance between the stimuli and its class is that of one or more inter-class, incorrect relations. This phenomenon coincides with the spike/decline in other relational metrics thanks to NE, also shown in Figure 12, and noted in Section 3. While the nodal distance spikes are insignificant in the broader course of an experiment, they highlight an interesting effect caused by the training structure.

These early findings also display the effects of NE on the agent's clip space and the RDT metrics, both at the end of training and throughout it. While the algorithm is suitable for its original intended purpose (i.e., generating transitivity and equivalence relations at the end of the training phase) within the purpose of EEPS (simulating the behavior of humans in MTS tasks), we wish to explore beyond this field in future projects at the potential of AI models which can perform equivalence responding without directly programming the ability to form symmetry or transitivity relations into thier architecture. There has been recent work in this area, with Carrillo and Betancort (2024) evaluating the equivalence responding ability of large-language models using MTS procedures, finding that these models are display equivalence responding behavior under a linear training structure, but cannot derive relations under non-linear designs. NE is a step in this direction, but there is an opportunity for an alternative approach that either alleviates these effects or incorporates aspects of RDT in its consolidation process, could be applied in its place. One potential approach could be to employ network embedding, where networks are visualized as vectorial data in a low-dimensional space, thus providing a clearer picture of their structure and supporting analysis methods such as clustering and similarity search (Nelson et al., 2019). For EEPS and Affinity, network embedding would allow for the representation of the higher-order properties in a two-dimensional space, as per Belisle and Dixon (2020b), or by incorporating the geometric designs presented in Belisle and Clayton (2021), where classes possess perimeters and areas based on their coherence.

However, the results are not without their limitations. These have been considered in framing the results as a proof-of-concept rather than a complete recreation of, and comparison with Cotter and Stewart (2023). Firstly, Cotter and Stewart utilized response latency in their study (the speed at which a user matched a sample stimulus to a comparison stimulus when presented). This was a reliable choice, given that prior studies had linked decreases in the speed of a participant's response to increases in the size of a class/nodal distance (Arntzen and Holth, 2000; Spencer and Chase, 1996). EEPS and other computational models are designed to simulate laboratory experiments in a fraction of the time it takes a human participant to do the same, and therefore, cannot provide a tangible response latency similar to the one measured by Cotter and Stewart. However, the initial insights provided by Affinity posit the mean transition probability as a reliable alternative.

The simulation of Cotter and Stewart (2023) has been limited to its first five phases, excluding the counterconditioning in phase 6. This is due to Affinity's lack of the necessary tools for measuring the resistance of individual relations. Based on Belisle and Dixon (2020b)'s definitions, relational mass should describe the resistance of a network. Affinity, in its current form, is only capable of calculating relational mass at a class level, and we are yet to implement tracking of the resistance of individual relations. Therefore, we decided to limit our demonstration to these first five phases, which would provide a picture of what insights can be obtained in Affinity and allow for an informal analysis of the first two predictions of Cotter and Stewart's study and the correlations between density and volume. Identifying the individual mass of relations in the agent's network would be a worthwhile addition to Affinity.

Another limitation of the demonstration is the inclusion of a mastery criterion. Each agent in the simulation was required to achieve a 0.9 (or 90%) mastery of the relations trained in each phase before progressing to the next stage. This is contrary to the original study, where participants were only required to achieve 14 out of 16 correct responses in the pre-training phase, and were only selected for analysis based on accuracy at various stages of the study. The mastery criterion in our version has resulted in the agents repeating each phase multiple times in our simulation (vs. the single phases for each participant), but this was the only realistic way to regulate the agents' performance over the five phases. Additionally, in their discussion, Cotter and Stewart note that a limitation of their study was that the density before phase 6 was not controlled, and could be done via a mastery criterion, which would improve its validity.

**Figure 13:**
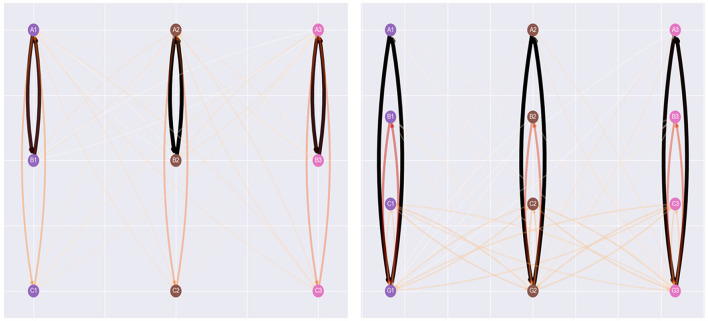
An example of the effects of two different training structures on the agent's clip space. Both graphs display Spencer and Chase (1996)'s study midway through the simulation, with the left utilizing a one-to-many setup (a single sample stimulus is trained to several comparison stimuli) vs. the right using a many-to-one setup (several sample stimuli are trained to a single comparison stimulus).

Despite the limitations, this paper has showcased several of Affinity's innovative features. The multi-threaded design, which allows users to run several versions of EEPS alongside each other, has been convenient in testing Affinity. This feature, combined with the graph network visualization, has been intuitive in understanding the effects of NE on the agent's clip space despite its tendency to become cluttered throughout the simulation. This use case is exemplified in Figure 13, illustrating how the graph visualization in Affinity can elucidate the effect of different training structures. The two graphs display one-to-many and many-to-one structures in the Spencer and Chase (1996) study, highlighting another practical application of Affinity. On the whole, the demonstrations provided in this report have highlighted the value of interpretable models of SE in developing a greater understanding of the underlying mechanisms and methodological effects of this paradigm.

Also, while these features have played a significant part in development and analysis, a few Affinity features have not been demonstrated or utilized heavily in this paper. Most notable is the relation editor interface, through which a user can modify the structure of an agent's clip space during an experiment. A novel concept in PS research, the ability to reduce or increase the strength of relations, could be utilized to appraise how neurological conditions affect a participant's performance. Studies such as Cowley et al. (1992) and Paranhos et al. (2018) have identified differences in performance on MTS procedures for individuals with brain injuries and strokes. Affinity could be a preliminary tool for ideating how relational networks are potentially altered under these circumstances.

As per Tovar et al. (2023)'s and Mofrad et al. (2020)'s suggestions, a significant step forward would be to introduce a generalization mechanism into EPS/EEPS, which could allow for modeling of compound stimuli in the agent's environment and, more significantly, the simulation of RFT. The generalization mechanism in PS would take the form of a wildcard clip in the agent's clip space that activates when two or more stimuli share a standard set of features (Melnikov et al., 2017). In the case of RFT, this wildcard clip would help identify the type of relation between the two stimuli, e.g., similarity or opposition. The development and testing of this proposed design would be more accessible via the visualizations provided in Affinity, which can clarify how the agent's clip space should be modified to introduce a broader range of relational responding.

Beyond implementing RFT, Affinity and EEPS would also benefit from several quality-of-life improvements. For example, the implementation interactive filtering tools to manage visual clutter–such, such as hiding edges below a threshold *h*-value in the graph network visualization and RDT measures, or generating dynamic subgraph views on specific stimuli, would enhance the analytics functions of the software. Other additions include keyboard shortcuts, heatmap interaction, result tables, and an interface for creating new experiments. A more significant overhaul would involve modifications to the model's design. For example, including multiple training and testing phases would provide a helpful addition in the simulation of the subset of classical and modern studies that utilize this structure. Alongside Cotter and Stewart (2023), another example is Pilgrim and Galizio (1995), which explored the reversal of baseline relations in adults. The study's six phases included various probes of transitivity and equivalence relations alongside training sections. Pilgrim and Galizio's study is pivotal as one of SE's first explorations of counterconditioning. It would be valuable to EEPS's accompanying classical study simulations collection. With Affinity, additional testing phases could be incorporated in the interface as additional tabs alongside the visualizations.

As discussed in Section 1, augmenting a pre-existing model of Stimulus Equivalence with RDT was an attractive prospect for several reasons. However, an alternative would be to design a simulation of RDT from the ground up. Edwards (2024) has outlined a novel application of RDT as part of their proposed neurosymbolic model of value alignment for large language models. By integrating RDT with the clustering method “Density-Based Spatial Clustering of Applications with Noise” (DBSCAN), a large language model, with its own relational network of stimuli, can identify which stimuli should be clustered together. This design is incorporated into a wider system by Edwards, but on its own, integrating RDT and DBSCAN clustering would provide a unique foundation for simulating RDT, including several of the emergent properties of high-mass networks, such as acceleration and gravity. Additionally, this approach could be applied in the context of word embeddings (Jang et al., 2016), where RDT can capture and provide insights with the relational properties of a corpus of text.

By working toward and integrating explainability into simulations of SE and RFT, an opportunity is presented for creating an observation-driven computational model that provides predictions of a human participant's behavior in real-time throughout an experiment. The factored design of Affinity would easily support an extension to map the agent's decision-making process to inputs from a human participant. This hybrid design would be akin to a digital twin, an approach for simulating real-world systems by creating a digital counterpart. Clinical and psychiatric applications have utilized digital twins for supporting clinical trials using participant information and data from follow-up visits (Das et al., 2023), and as an approach for early diagnosis and risk assessment (Alimour and Alrabeei, 2024). These use cases highlight the potential of a digital twin system for MTS-based experiments and potentially process-based therapy.

Beyond computational simulations, Contextual Behavioral Science has the potential to inform and inspire future work in explainable AI. There is a growing body of literature investigating integrations between the two fields. For example, Edwards et al. (2022) have created a framework using RFT to abstract background knowledge in category learning models, where the theory is integrated with connectionist models which support the system in achieving arbitrary and non-arbitrary relational responding. Additionally, there have been several contributions exploring SE and RFT in the context of Artificial General Intelligence (Johansson, 2019; Johansson and Lofthouse, 2023), including implementations of relational and equivalence responding in logical systems geared toward these all-encompassing models. From these perspectives, Affinity represents a tangible contribution that, while situated in the modeling paradigms of SE, acts as a step forward for synthesizing the two fields.

## Data Availability

Publicly available datasets were analyzed in this study. This data can be found at: https://github.com/jamosully/EEPS-Visualizations.
